# Intergenerational Education and Late-life Cognitive Decline among Latinos and Non-Hispanic Whites

**DOI:** 10.1093/geroni/igab046.3308

**Published:** 2021-12-17

**Authors:** Erika Meza, Yea-Hung Chen, Isabel Allen, Hector Gonzalez, M Maria Glymour, Jacqueline Torres

**Affiliations:** 1 University of California, San Francisco, San Francisco, California, United States; 2 University of California, San Diego, San Diego, California, United States; 3 University California San Francisco, San Francisco, California, United States

## Abstract

Latinos face a growing burden of Alzheimer’s Disease and related dementia (ADRD). Although education has been established as a strong predictor of ADRD, evidence to date is primarily for non-Latino cohorts. Few studies have assessed the relationship between intergenerational education and one’s cognitive decline. Using the US Health and Retirement Study (N=20,860) we evaluated the joint effect of parental and own educational attainment on immediate and delayed verbal memory scores (range 0-10) from 1998 to 2016. The exposure was a 4-category variable based on parents’ (highest of mother’s or father’s) and participant’s own high school attainment: first-generation (parents’ education <12; own ≥12); multi-generation (both ≥12: REF); neither graduated high school (both <12) and parent(s) graduated high school but not respondent (parents ≥12; own <12). Linear mixed effects models with subject-specific random intercepts and random slopes were stratified by race/ethnicity and tested for a 3-way interaction term (exposure x Latino x time). Models controlled for age, sex, place of birth and retest effects. Baseline verbal memory scores did not differ for first-generation compared to multi-generation high school graduates. Verbal memory decline was faster for first- compared to multi-generation high school graduates among non-Hispanic whites (e.g., β=-0.04; 95% CI: -0.05, -0.03, delayed verbal recall); among Latinos, first and multi-generation high school graduates had similar rates of decline (e.g. β=0.00; 95% CI: -0.03, 0.04, delayed verbal recall; p<0.001 for three-way interaction). Our findings suggest social and economic policies that facilitate educational achievement, particularly for important population subgroups, may reduce ADRD risk.

